# Self-Reported Clinical Practice of Small Animal Cardiopulmonary Resuscitation and Compliance With RECOVER Guidelines Among Veterinarians in Eight Western European Regions

**DOI:** 10.3389/fvets.2022.919206

**Published:** 2022-07-14

**Authors:** Simon P. Hagley, Anne Kruppert, Rodolfo Oliveira Leal, José Carlos Pizarro del Valle, Claudia Iannucci, Imke Hennink, Ludivine Boiron, Sabrina N. Hoehne

**Affiliations:** ^1^Emergency and Critical Care Department, Vets Now Referrals, Manchester, United Kingdom; ^2^Division of Anaesthesia and Analgesia, Department of Clinical Veterinary Science, Vetsuisse Faculty, University of Bern, Bern, Switzerland; ^3^CIISA Centre for Interdisciplinary Research in Animal Health, Faculty of Veterinary Medicine, University of Lisbon, Lisbon, Portugal; Associate Laboratory for Animal and Veterinary Sciences (AL4AnimalS); ^4^, Small Animal Hospital, School of Veterinary Medicine, College of Medical Veterinary and Life Sciences, University of Glasgow, Glasgow, United Kingdom; ^5^Division of Small Animal Emergency and Critical Care, Department of Clinical Veterinary Science, Vetsuisse Faculty, University of Zurich, Zurich, Switzerland; ^6^Division of Small Animal Emergency and Critical Care, Department of Clinical Veterinary Science, Vetsuisse Faculty, University of Bern, Bern, Switzerland; ^7^Department of Emergency and Critical Care, Clinique Vétérinaire Languedocia, Montpellier, France; ^8^Department of Veterinary Clinical Sciences, College of Veterinary Medicine, Washington State University, Pullman, WA, United States

**Keywords:** cardiopulmonary resuscitation, Europe, guidelines, RECOVER, compliance

## Abstract

**Introduction:**

The objective of this study was to assess whether small animal veterinarians across Western Europe are compliant with the 2012 cardiopulmonary resuscitation (CPR) guidelines by the Reassessment Campaign on Veterinary Resuscitation (RECOVER).

**Methods:**

A previously published online questionnaire from Switzerland was adapted and translated into 7 languages, corresponding to national languages in Austria, France, Germany, Ireland, Italy, Liechtenstein, Netherlands, Portugal, Spain, and the United Kingdom. The survey was distributed via respective national veterinary organizations and social media outlets. A subset of questions was analyzed to evaluate respondent demographics, RECOVER guideline awareness, and to allocate composite compliance scores for CPR preparedness, basic life support (BLS) and advanced life support (ALS). Percentages of group total (95% confidence interval) were calculated. Multivariable logistic regression was used to evaluate the effects of region of practice, gender, age, specialty training, and guideline awareness on compliance. Odds ratios (95% confidence interval) were generated and significance set at *P* < 0.05.

**Results:**

Nine-hundred and thirty respondents were included in analysis. Awareness of and compliance with RECOVER guidelines varied widely across regions. Compliance with all assessed RECOVER guideline recommendations was highest in Germany/Austria [14% (7- 27%)] and lowest in France and Portugal [0% (0–3%)]. CPR preparedness compliance was higher in participants aware of RECOVER guidelines [OR 10.1 (5.2-19.5)], those practicing in Germany/Austria [OR 4.1 (1.9–8.8)] or UK/Ireland [OR 2.2 (1.3–3.7)], and lower in those practicing in Portugal [OR 0.2 (0.1–0.9)]. Specialty training [OR 1.8 (1.1–2.9)], guideline awareness [OR 5.2 (3.2–8.6)], and practice in Germany/Austria [OR 3.1 (1.5–6.5)], UK/Ireland [OR 2.6 (1.7–4.1)], or the Netherlands [OR 5.3 (2.0–14.2)] were associated with increased BLS compliance. ALS compliance was higher in participants with guideline awareness [OR 7.0 (2.9–17.0)], specialty training [OR 6.8 (3.8–12.1)], those practicing in Germany/Austria [OR 3.5 (1.3–9.6)], UK/Ireland [OR 4.0 (1.9–8.3)], or Spain [OR 3.2 (1.2–8.3)] and in younger survey participants [OR 0.9 (0.9–1.0)].

**Conclusions:**

Awareness and compliance with RECOVER guidelines varied widely among countries surveyed, however overall compliance scores in all countries were considered low. Further research may highlight factors surrounding poor guideline awareness and compliance so targeted efforts can be made to improve veterinary CPR in Europe.

## Introduction

Evidence-based clinical practice guidelines for the conduction of cardiopulmonary resuscitation (CPR) in dogs and cats have only been available to the small animal practitioner for the past ten years (1). Prior to the publication of guideline recommendations, there was no standardization of small animal CPR, and suggestions for CPR techniques were largely extrapolated from human medicine (2–5). Accordingly, an international, internet-based survey conducted in 2008 demonstrated that clinical CPR practice amongst small animal practitioners varied widely (6).

In 2012, the Reassessment Campaign on Veterinary Resuscitation (RECOVER) conducted a systematic evaluation of evidence and published the first small animal CPR consensus guidelines (1). The taskforce reviewed five clinically relevant domains surrounding small animal CPR, which included preparedness and prevention measures, basic life support (BLS), advanced life support (ALS), monitoring, and post-cardiac arrest care. This review ultimately allowed the formulation of clinical practice guidelines (1, 7–11). A veterinary study assessing changes in small animal CPR teaching before and after publication of the RECOVER guidelines demonstrated a change in clinical practice subsequent to inclusion of these guidelines in CPR training (12). Furthermore, implementing RECOVER guideline recommendations in clinical practice was recently shown to significantly improve veterinary CPR patient outcomes (13). This supports the strong evidence in the human literature that CPR guideline compliance results in more positive patient outcomes (14–16).

Several factors are important to allow CPR guidelines to alter clinical practice and improve patient outcomes. These include awareness of current CPR recommendations, training of the veterinary team in accordance with guidelines, and compliance with the guideline-instructed teaching such that they are effectively implemented in clinical CPR situations. A follow up survey conducted in 2017 investigated the worldwide compliance of small animal practice with RECOVER guidelines and found that while awareness of these guidelines is high in veterinary specialists, it remained insufficient in the general practitioner population (17).

Less is known specifically about awareness of and compliance with RECOVER guidelines among European veterinarians as only 24% of respondents in the worldwide study were based in Europe (17). In one previous study surveying veterinarians in Switzerland, only 8% of general practitioner respondents reported to have heard of the RECOVER clinical practice guidelines and self-reported CPR practice amongst Swiss veterinarians was assessed to largely not be in agreement with current recommendations (18). This may differ in other European countries and further information on regional CPR practice is needed to develop strategies to increase guideline awareness and compliance amongst European veterinary professionals.

The aims of this study were therefore to determine awareness of, and compliance with RECOVER guideline recommendations across Western Europe, to identify factors associated with guideline awareness and compliance, and to report potential differences among surveyed countries.

## Materials and Methods

### Survey Generation and Distribution

Data was obtained in the form of an online questionnaire developed, along with the study protocols, at the Vetsuisse Faculty of the University of Bern, Switzerland. All distributed materials to recruit survey respondents from participating countries declared the research as a project of the University of Bern, Switzerland and the internal ethics review board at the Vetsuisse Faculty, University of Bern waived the need for ethical approval. Participation in this study was strictly voluntary, no incentive was offered for participation, and survey responses were collected anonymously. By choosing to participate, each respondent was consenting to use of their answers for the purposes of this study.

To investigate the compliance of small animal CPR conduction with current RECOVER guidelines in Western Europe, collaborators located in or with active contacts to national veterinary associations were identified for the countries of Austria (AK), England (SPH), France (LB), Germany (AK), Ireland (SPH), Italy (CI), Liechtenstein (AK), Netherlands (IH), Northern Ireland (SPH), Portugal (RL), Scotland (SPH), Spain (JCP), and Wales (SPH). Collaborators facilitated questionnaire cross-cultural adaptation and translation to the national language(s), and survey distribution for their assigned country(ies).

This study was a follow up study to a national survey investigating the clinical practice of small animal CPR and compliance with RECOVER guidelines in Switzerland previously conducted by two investigators (AK and SNH) of this group (18). The questionnaire used to survey veterinarians of individual countries was comprised of the same questions and format used in the Swiss study, which were initially adapted from an international survey on small animal CPR conduction (17, 18). Questionnaires contained up to 49 questions covering respondent demographics, work environment, opinions on small animal CPR, familiarity with the RECOVER guidelines, and specific questions pertaining to CPR training frequency, preparedness measures, equipment available in the workplace, and BLS and ALS techniques routinely employed. Survey questions included single and multiple-answer multiple-choice formats, Likert's and slider scale questions, and numerical and categorical ranking questions. For select questions offering an “other” answer choice, a free text field was provided. The remainder of the questions were closed-ended (see full English questionnaire in Supplementary Data 1).

**Figure 1:**
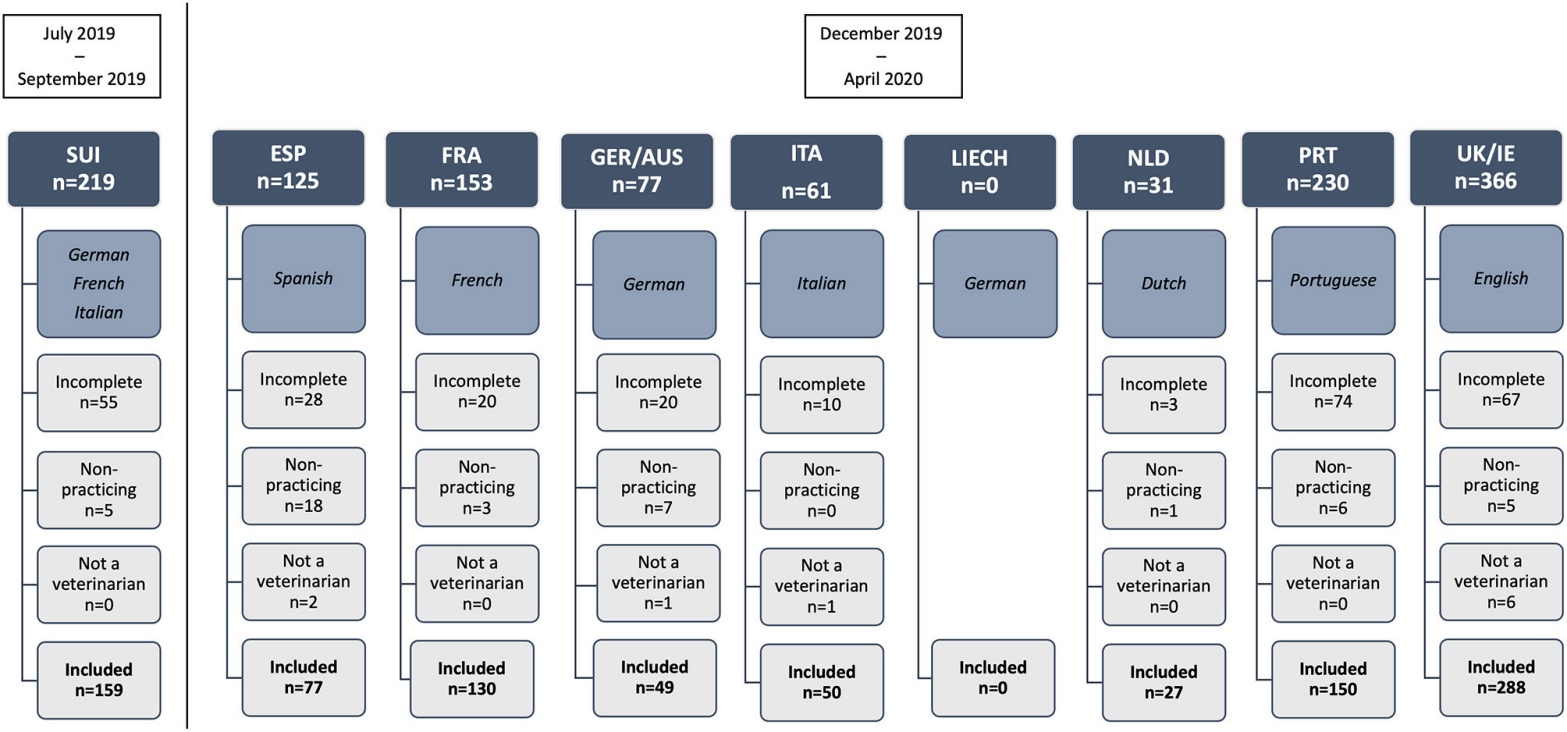
Flow diagram showing language of survey dissemination and number of survey respondents by European region, and reason for inclusion or exclusion in the final study. ESP, Spain; FRA, France; GER/AUS, Germany/Austria; ITA, Italy; LIECH, Liechtenstein; NLD, Netherlands; PRT, Portugal; SUI, Switzerland; UK/IE, England/Northern Ireland/Scotland/Wales/Ireland.

Finalized questionnaires were uploaded to a commercially available, internet-based survey development and administration tool (Survey Monkey^®^, www.surveymonkey.com). Seven specific survey links were generated for each survey language with some questionnaires adapted to enable respondents to select their country of origin if the same language was shared among multiple countries. For multi-lingual countries, survey links containing all relevant languages were distributed as described in Figure 1. The survey platform collector options were set to allow participation in the survey from the same device only once using authentication cookies. Respondents were expected to require approximately 10 minutes to complete the full survey. All surveys were uploaded and ensured to be functional in December 2019 and advertisement for survey participation took place between December 2019 and April 2020 in Austria, England, France, Germany, Ireland, Italy, Liechtenstein, Netherlands, Northern Ireland, Portugal, Scotland, Spain, and Wales. Each collaborator was responsible for the distribution of their survey link(s) through regional veterinary associations and practices and a list of outlets used for recruitment can be found in Supplementary Table 1. The distribution of reminder e-mails and posts was at the discretion of the individual collaborator and not standardized amongst countries. All surveys closed on April 10^th^, 2020 and raw data was downloaded from the survey collector homepage on April 16^th^, 2020. A survey of Swiss veterinarians was not repeated, instead data from the initial survey was included in this analysis (18). Links to German, French, and Italian surveys were initially distributed to Swiss veterinarians in July 2019, a reminder sent in August 2019, and the surveys closed at the end of September 2019 (18).

### Allocation of CPR Guideline Compliance Scores

For the purpose of this study, we only extracted and analyzed questions on respondent demographics, those that reflect specific RECOVER guideline recommendations in the areas of CPR preparedness, BLS execution, and ALS equipment and execution, and those that investigated respondent awareness and perception of the RECOVER clinical guidelines (see abbreviated questionnaire containing the questions analyzed in Supplementary Data 2). The remainder of the data set describing additional aspects of CPR conduction amongst European countries will be analyzed separately and published elsewhere.

Eleven of the questions analyzed herein covered respondent characteristics such as country of practice, gender, age, post-graduate specialty training, current career status, current clinical environment, case load, and whether or not CPR was routinely offered and practiced. For the purpose of this study, veterinarians were considered to have undergone specialty training if they were currently enrolled in a residency program, held diplomate status in a veterinary specialty discipline, or were residency trained and board eligible to obtain such diplomate status. Ten questions gathered information on CPR preparedness measures in place in respondents' work environments, BLS performance, and ALS performance. Lastly, the final three questions asked respondents' opinions on the importance of CPR knowledge, self-assessment of CPR skills, and awareness and importance of veterinary CPR guidelines. Survey responses were included in the analysis if they originated from veterinarians currently practicing clinical veterinary medicine and if all questions to determine CPR guideline compliance scores were answered. Responses were excluded from analysis if they stemmed from veterinary students or non-practicing veterinarians or veterinary support staff, if respondents were not practicing in the country for which the survey was intended, or if insufficient data was available to evaluate the respondents' practice characteristics or derive CPR guideline compliance scores. As previously described by Gillespie et al., compliance composite scores were derived for the following three aspects: CPR preparedness, BLS execution, and ALS execution of the RECOVER clinical CPR guidelines (17). Survey participants of every country were considered compliant in CPR preparedness if they had participated in CPR training within 6 months of survey completion, if they displayed CPR cognitive aids in their practice (CPR algorithm and emergency drug dosing chart), and if they stocked and regularly maintained a crash cart (1, 17). BLS conduction was considered to be in compliance with RECOVER guidelines if knowledge of recommended chest compression rates of 100 to 120 compressions/min and positive pressure ventilation rates of 6–15 breaths/min for both dogs and cats was demonstrated (1, 17). ALS compliance was defined as survey respondents having access to vasopressors (epinephrine and/or vasopressin), atropine, anti-arrhythmic agents (amiodarone and/or lidocaine), sodium bicarbonate, *not* using intravascular volume expansion routinely, having access to an electrical defibrillator, and using electrocardiogram (ECG) and end-tidal carbon dioxide (EtCO_2_) monitoring routinely (1, 17). Lastly, based on the individual scores for CPR preparedness compliance, BLS compliance, and ALS compliance, a composite score was created to assess overall RECOVER guideline compliance. Compliance with all aspects of RECOVER guideline recommendations was achieved if survey respondents were compliant with guideline recommendations in all three areas of CPR preparedness, BLS, and ALS.

### Statistical Analyses

Responses from the collector homepage were downloaded and transferred into a commercial computer spread sheet program (Microsoft Excel^®^). Available responses were reviewed and questions not relevant to the aims of this study removed. Subsequently, responses were excluded from analysis if the remaining questions did not contain complete demographic information, sufficient answers to calculate RECOVER compliance scores, or if respondents met other exclusion criteria as outlined above. Based on small numbers of respondents from several of the 14 surveyed countries, responses from multiple countries were combined into eight Western European regions for statistical analysis, if they shared the same language. Percentages of group total and 95% confidence intervals (CI) were calculated for categorical data and results are presented as percentage (95% CI). Continuous data, including Likert-scale scores, were tested for normality using the Shapiro-Wilk test and by examining normal plots. Normally distributed data are presented as mean +/− SD and non-normally distributed data as median (interquartile range; IQR). Factors associated with RECOVER guideline awareness, CPR preparedness compliance, BLS compliance, ALS compliance, and overall RECOVER compliance were assessed by multivariable binomial logistic regression analyses using a backwards stepwise approach. Five logistic regression models were generated for the above dependent outcomes of interest. Independent variables evaluated for association with CPR preparedness, BLS, ALS, and overall RECOVER compliance included the eight Western European Regions, as well as factors likely to confound guideline compliance such as gender, age, specialty training, and guideline awareness. Independent variables included in the model generated for guideline awareness included the eight Western European Regions, as well as gender, age, and specialty training. Odds ratios with 95% CI and associated *P*-values were generated for the effect of each variable on guideline awareness or compliance and the Hosmer-Lemeshow goodness of fit test was used to assess model fit. Statistical significance was set at <0.05.

All analyses were performed using commercially available statistical programs [Prism 9, GraphPad Software, La Jolla, CA, U.S.A. and SPSS Statistics for Windows, version 28.0.1.1. (14), Chicago, IL, U.S.A.].

## Results

### Western European Regions and Respondent Characteristics

A total of 1,262 responses were received from the 14 surveyed countries, of which 930 responses were included in analysis. Three hundred and thirty-two responses were excluded from analysis and details are provided in Figure 1. Of the included responses, data of respondents practicing in England (*n* = 237), Ireland (*n* = 3), Northern Ireland (*n* = 3), Scotland (*n* = 37), and Wales (*n* = 8) were combined as the geographic region of “United Kingdom/Ireland (UK/IE)” for analysis. Responses from Germany (*n* = 45) and Austria (*n* = 4) were combined as the geographic region of “Germany/Austria (GER/AUS)” for analysis. Responses from Spain (ESP; *n* = 77), France (FRA; *n* = 130), Italy (ITA; *n* = 50), Netherlands (NLD; *n* = 27), Portugal (PRT; *n* = 150), and Switzerland (SUI; *n* = 159) were not combined for statistical analysis and each country corresponds to a separate geographical region. No responses from veterinarians practicing in Liechtenstein were received (*n* = 0).

Respondent population characteristics, professional status, and information on clinical practice environment by region are summarized in Table 1.

**Table 1:** Demographic information and clinical environment characteristics of survey respondents across Western Europe by region.

	**ESP**	**FRA**	**GER/AUS**	**ITA**	**NLD**	**PRT**	**SUI**	**UK/IE**
**Respondents per group**	***n* = 77**	***n* = 130**	***n* = 49**	***n* = 50**	***n* = 27**	***n* = 150**	***n* = 159**	***n* = 288**
Age [years, median (IQR range)]	32 (29–38)	36 (31–45)	33 (29–41)	35 (31–40)	35 (31–40)	33 (29–42)	41 (32–53)	33 (29–40)
Female respondents	64 (53–74)	66 (58–74)	73 (60–84)	62 (48–74)	82 (63–92)	84 (77–89)	73 (66–79)	71 (65–76)
Board certified/ residency trained specialists or resident trainee	20 (12–30)	4 (2–9)	43 (30–57)	4 (1–14)	4 (0–18)	0 (0–3)	18 (13–24)	22 (18–27)
Respondents with > 5 veterinarians in practice	64 (53–74)	36 (28–45)	84 (71–92)	82 (69–90)	59 (41–76)	35 (28–43)	87 (81–91)	67 (61–72)
Respondents treating >50% small animal patients	97 (91–100)	93 (87–96)	100 (93–100)	100 (93–100)	100 (88–100)	100 (98–100)	90 (84–94)	100 (98–100)
Number of treated patients per veterinarian per day > 10 patients	34 (24–45)	71 (63–78)	57 (43–70)	66 (52–78)	78 (59–89)	37 (30–45)	65 (58–72)	62 (56–68)
Respondents with caseload ≥ 50 % emergencies per day	13 (7–22)	5 (2–10)	8 (3–19)	12 (6–24)	30 (16–49)	23 (17–30)	8 (4–13)	21 (16–26)
Respondents performing CPR ≥ 6 times per year	42 (31–53)	28 (21–36)	57 (43–70)	72 (58–83)	22 (11–41)	30 (23–38)	18 (13–25)	19 (15–24)
Respondents with resuscitation team of ≥ 4 people	39 (29–50)	12 (8–19)	61 (47–74)	36 (24–50)	11 (4–28)	84 (77–89)	26 (20–33)	50 (45–56)

*Data are presented as percentage respondents per region (95% Confidence Interval). ESP, Spain; FRA, France; GER/AUS, Germany/Austria; ITA, Italy; IQR, Interquartile range; NLD, Netherlands; PRT, Portugal; SUI, Switzerland; UK/IE, England/Northern Ireland/Scotland/Wales/Ireland*.

### Perception of CPR and Awareness of RECOVER Guidelines

The majority of respondents from all regions offer CPR in their practice to all or a subset of patients. The proportion of respondents that perform CPR in their practice in ESP was 95% (87–98%), in FRA was 95% (89–97%), in GER/AUS was 94% (84–98%), in ITA was 100% (93–100%), in the NLD was 100% (88–100%), in PRT was 96% (92–98%), in SUI was 93% (88–96%), and in the UK/IE was 99% (98–100%). On a scale of 0 (not essential) to 100 (absolutely essential), the majority of respondents perceived CPR to be an essential skill in small animal veterinary medicine. The median (IQR) ascribed importance by respondents in ESP was 100 (93–100), in FRA was 84 (71–99), in GER/AUS was 100 (89–100), in ITA was 100 (98–100), in the NLD was 80 (70–100), in PRT was 100 (91–100), in SUI was 80 (50–100), and in the UK/IE was 99 (85–100).

In half of the surveyed regions, the majority of respondents reported to be aware of the RECOVER CPR guidelines. When asked whether they adhere to the RECOVER guidelines in clinical practice, fewer respondents than are aware of guideline existence responded with yes in the majority of regions. In the NLD, all respondents aware of guideline existence reported clinical adherence, while in GER/AUS, two more respondents than are aware of the guidelines responded to practice in adherence with their recommendations. The proportion of respondents with awareness of the RECOVER guidelines, and their reported rates of practice in accordance with these recommendations are presented in Table 2 and factors statistically significantly associated with RECOVER guideline awareness in Table 3.

**Table 2:** Self-reported and author-ascribed scores of compliance with, and awareness of the RECOVER guidelines in respondents from 8 Western European regions.

	**ESP**	**FRA**	**GER/AUS**	**ITA**	**NLD**	**PRT**	**SUI**	**UK/IE**
**Respondents per group**	***n* = 77**	***n* = 130**	***n* = 49**	***n* = 50**	***n* = 27**	***n* = 150**	***n* = 159**	***n* = 288**
Awareness of RECOVER	64 (53–74)	16 (11–23)	71 (58–82)	76 (63–86)	33 (19–52)	26 (20–34)	24 (18–31)	58 (53–64)
Self–reported compliance with RECOVER	61 (50–71)	9 (5–15)	76 (62–85)	48 (35–62)	33 (19–52)	18 (13–25)	21 (15–28)	55 (49–61)
Ascribed overall compliance	1 (1–7)	0 (0–3)	14 (7–27)	0 (0–7)	0 (0–13)	0 (0–3)	1 (0–5)	5 (3–8)
Ascribed compliance with CPR preparedness	13 (7–22)	2 (1–7)	35 (23–49)	4 (1–13)	4 (0–18)	1 (0–5)	7 (4–12)	20 (16–25)
Ascribed compliance with BLS execution	13 (7–22)	4 (2–9)	33 (21–47)	12 (6–24)	26 (13–45)	4 (2–9)	9 (6–15)	22 (18–27)
Ascribed compliance with ALS execution	14 (8–24)	0 (0–3)	22 (13–36)	2 (0–10)	4 (0–18)	0 (0–3)	6 (3–10)	16 (12–20)
Ascribed compliance with ALS execution (excluding access to a defibrillator)	29 (20–39)	3 (1–8)	24 (15–38)	10 (4–21)	7 (1–23)	1 (0–5)	6 (3–11)	23 (18–28)

*Data are presented as percentage respondents per region (95% Confidence Interval). ALS, Advanced Life Support; BLS, Basic Life Support; ESP, Spain; FRA, France; GER/AUS, Germany/Austria; ITA, Italy; NLD, Netherlands; PRT, Portugal; RECOVER, Reassessment Campaign on Veterinary Resuscitation; SUI, Switzerland; UK/IE, England/Northern Ireland/Scotland/Wales/Ireland*.

**Table 3:** Factors significantly associated with RECOVER guideline awareness and ascribed compliance with CPR preparedness, BLS execution, ALS execution, and overall RECOVER compliance in multivariable logistic regression analyses.

**Variable**	**OR**	**95 % CI lower**	**95 % CI upper**	***p*-value**
**RECOVER guideline awareness**
UK/IE	3.614	2.479	5.268	<0.001
GER/AUS	4.442	2.161	9.130	<0.001
ITA	10.276	5.049	20.915	<0.001
ESP	4.156	2.386	7.240	<0.001
Age	0.960	0.944	0.977	<0.001
Specialist	6.317	3.809	10.476	<0.001
**Overall recover compliance**
UK/IE	5.039	1.376	18.456	0.015
GER/AUS	10.496	2.445	45.060	0.002
Specialist	6.569	2.480	17.403	<0.001
Awareness	9.512	1.216	74.407	0.032
**Preparedness compliance**
UK/IE	2.152	1.253	3.699	0.006
GER/AUS	4.078	1.899	8.765	<0.001
PRT	0.216	0.050	0.942	0.041
Awareness	10.071	5.190	19.540	<0.001
**BLS compliance**
UK/IE	2.624	1.664	4.136	<0.001
GER/AUS	3.134	1.513	6.494	0.002
NLD	5.286	1.967	14.208	<0.001
Specialist	1.763	1.085	2.864	0.022
Awareness	5.204	3.162	8.566	<0.001
**ALS compliance**
UK/IE	3.997	1.898	8.331	<0.001
GER/AUS	3.546	1.309	9.603	0.013
ESP	3.203	1.231	8.337	0.017
Age	0.961	0.923	1.000	0.048
Specialist	6.815	3.834	12.115	<0.001
Awareness	6.968	2.871	16.913	<0.001

*Data are presented as Odds ratios (OR) with 95% confidence interval (95% CI). P-values of < 0.05 are considered statistically significant. ALS, Advanced Life Support; BLS, Basic Life Support; ESP, Spain; GER/AUS, Germany/Austria; ITA, Italy; NLD, Netherlands; PRT, Portugal; RECOVER, Reassessment Campaign on Veterinary Resuscitation; UK/IE, England/Northern Ireland/Scotland/Wales/Ireland*.

### Overall RECOVER Compliance

**Figure 2:**
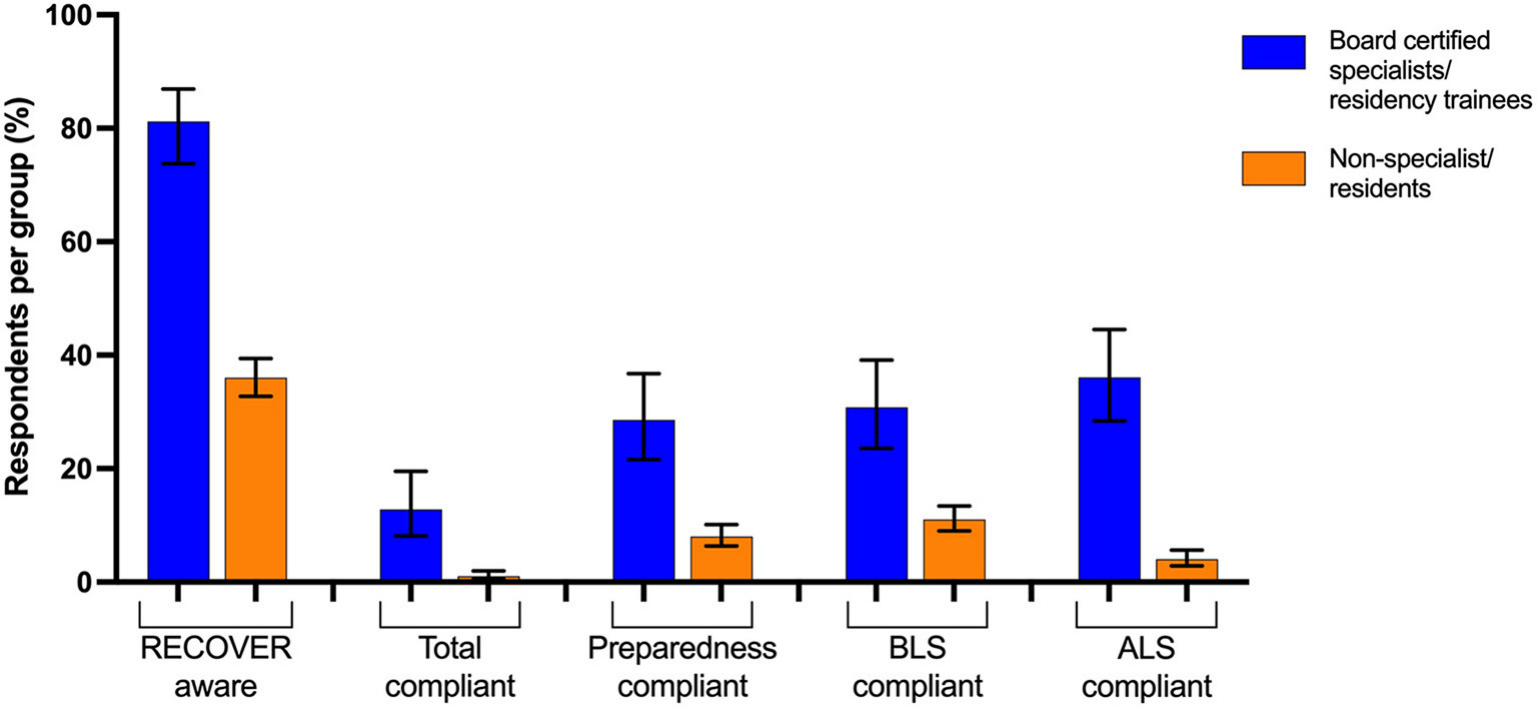
RECOVER guideline compliance and awareness of board-certified specialists or residency trainee respondents (*n* = 135) and respondents without residency training (*n* = 795) from 8 combined Western European regions. BLS, Basic Life Support; ALS, Advanced Life Support.

CPR practices reported by the majority of respondents in all European regions were not in compliance with the recommendations made by the RECOVER guidelines in all areas of CPR preparedness, BLS execution, and ALS execution (Table 2). Factors statistically significantly associated with overall RECOVER compliance are presented in Table 3. Board-certified or eligible specialists and residency trainees comprised 135 [15% (12–17%)] of total respondents across surveyed regions and specialty training was significantly associated with BLS, ALS, and overall RECOVER compliance (Table 3; Figure 2).

### CPR Preparedness Compliance

**Figure 3:**
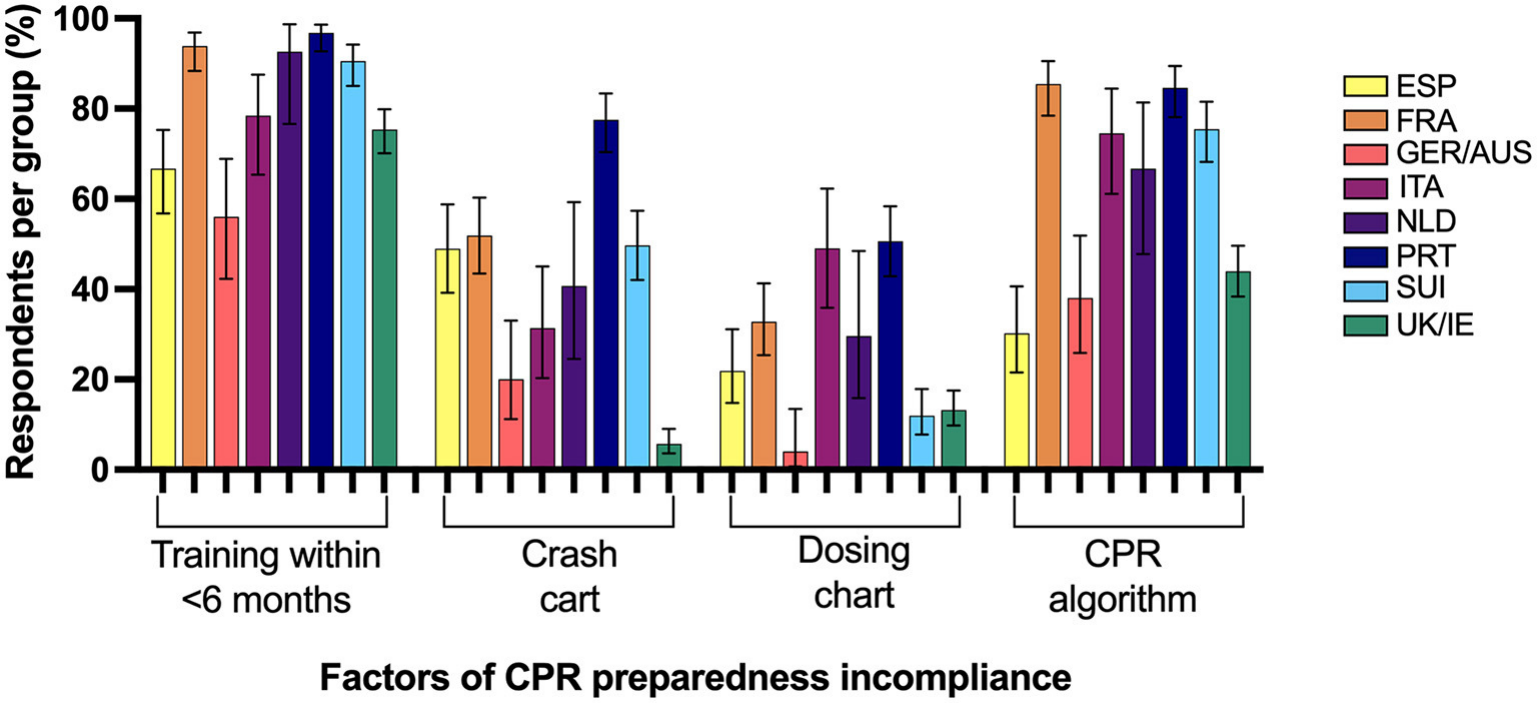
Percentage of respondents from 8 Western European regions incompliant with cardiopulmonary resuscitation (CPR) preparedness RECOVER guidelines and the associated factors for failed compliance. ESP, Spain; FRA, France; GER/AUS, Germany/Austria; ITA, Italy; NLD, Netherlands; PRT, Portugal; SUI, Switzerland; UK/IE, England/Northern Ireland/Scotland/Wales/Ireland.

The majority of respondents in all surveyed European regions were incompliant with RECOVER recommended CPR preparedness measures as shown in Table 2. Factors associated with CPR preparedness compliance are presented in Table 3 and those resulting in preparedness incompliance per region are shown in Figure 3.

### Basic Life Support Compliance

**Figure 4:**
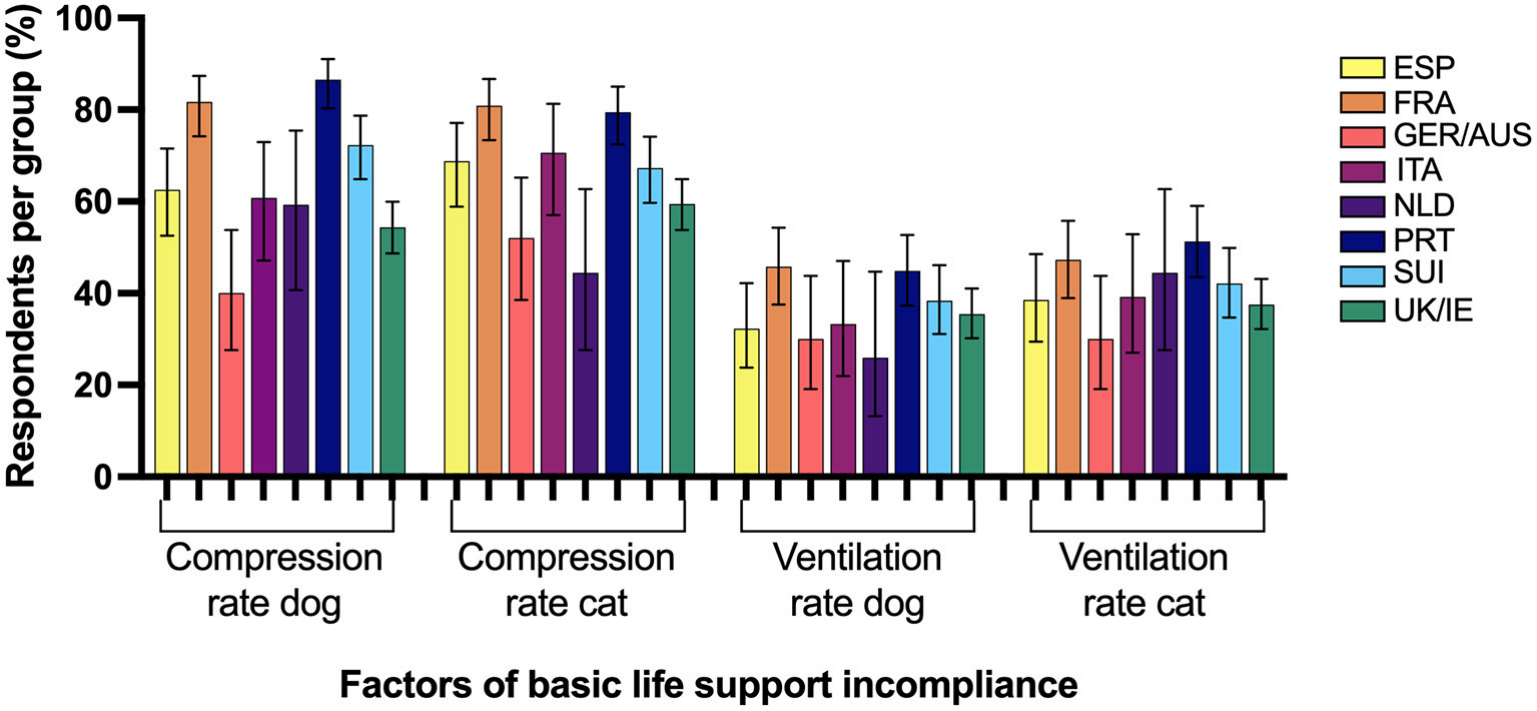
Percentage of respondents from 8 Western European regions incompliant with basic life support RECOVER guidelines and the associated factors for failed compliance. ESP, Spain; FRA, France; GER/AUS, Germany/Austria; ITA, Italy; NLD, Netherlands; PRT, Portugal; SUI, Switzerland; UK/IE, England/Northern Ireland/Scotland/Wales/Ireland.

Self-reported practice in compliance with all aspects of RECOVER BLS recommendations was present in a minority of responses in all surveyed regions as presented in Table 2. Factors associated with BLS compliance are presented in Table 3 and those contributing to BLS incompliance per region can be found in Figure 4.

### Advanced Life Support Compliance

**Figure 5:**
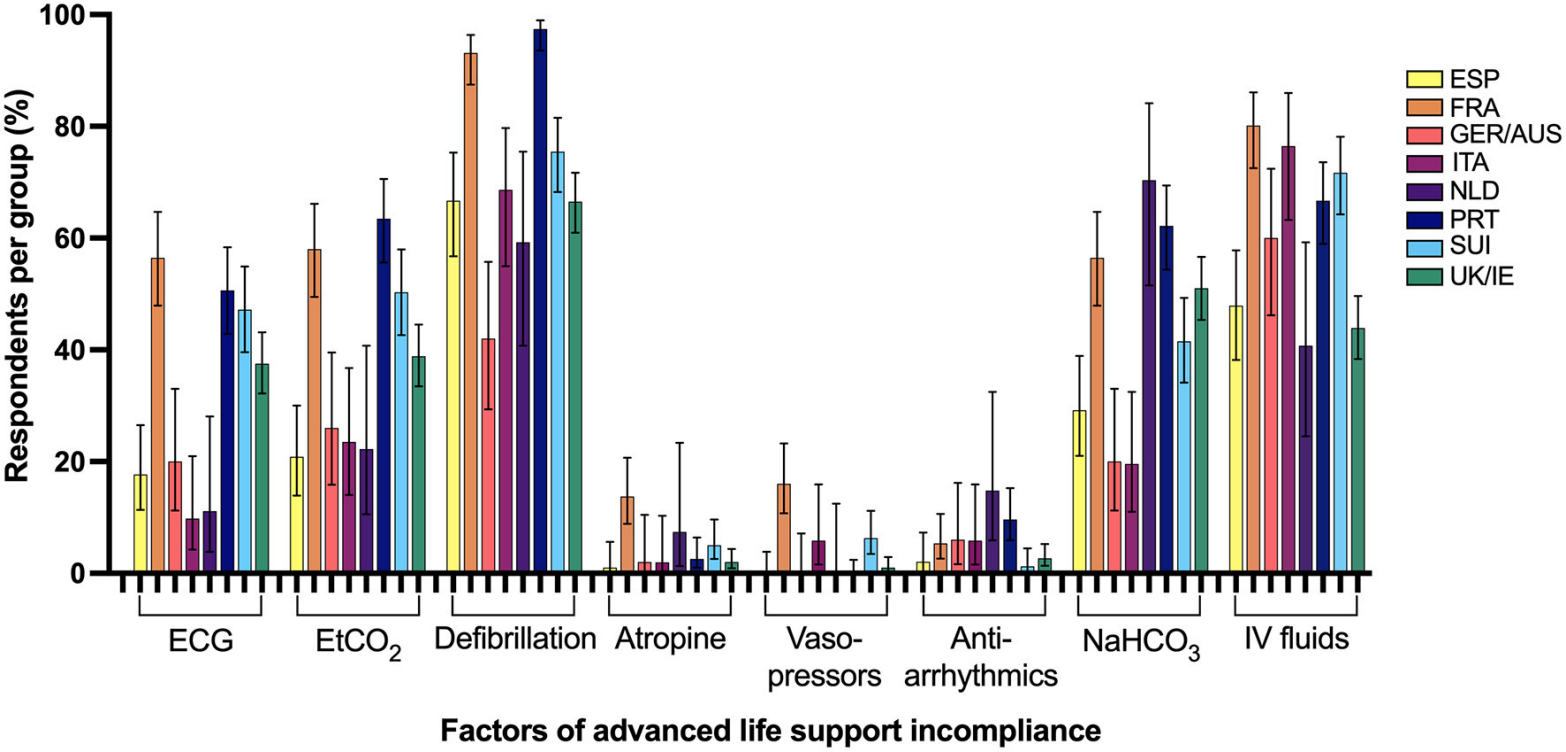
Percentage of respondents from 8 Western European regions incompliant with advanced life support RECOVER guidelines and the associated factors for failed compliance. ECG, Electrocardiogram; ESP, Spain; EtCO_2_, End-tidal carbon dioxide; FRA, France; GER/AUS, Germany/Austria; ITA, Italy; NaHCO_3_, sodium bicarbonate; NLD, Netherlands; PRT, Portugal; SUI, Switzerland; UK/IE, England/Northern Ireland/Scotland/Wales/Ireland; IV, Intravenous.

A minority of respondents in all surveyed regions were compliant with all RECOVER ALS recommendations (Table 2) and factors contributing to ALS incompliance are summarized in Figure 5. Factors statistically significantly associated with ALS compliance can be found in Table 3. When the requirement for access to an electrical defibrillator was excluded from the assignment of ALS compliance, a notable increase in RECOVER guideline compliance was seen in almost all regions.

## Discussion

This study reports the awareness of veterinary CPR guidelines and self-reported clinical practice of small animal CPR in eight regions of Western Europe. Although inter-regional variation exists, a significant number of respondents to the survey were unaware of the RECOVER guidelines and in general, the self-reported CPR performance across regions was not in compliance with them.

The overall number of participants in the survey is disappointing when considering in 2019 the number of veterinary practitioners registered in each surveyed country ranged from 2,949 (in Switzerland) to ~41,000 (in Germany) (19). In 2019 the Federation of Veterinarians of Europe reported the demographics of the European veterinary population, listed by country, and shows the demographic data presented in our study to be reflective of the veterinary population in clinical practice within each region (19). Median age of respondents was similar to previously published veterinary CPR performance studies, with the average respondent being aged between 32 and 41 years old (17, 18, 20). The most commonly reported age group for European veterinarians in a large demographic survey is 30–44 years (19). The sex distribution of respondents was reflective in all regions of the reported female bias of 58% in European veterinarians (19).

Almost all respondents treated predominantly small animals though there was variation between European regions in number of veterinarians in the practice and number of patients treated per day. Additionally, only a small proportion of respondents had a high emergency caseload and only the minority performed CPR more than 6 times per year. The potential bias this information imparts upon our results must be considered, as it is reasonable to assume veterinarians outside of the field of emergency and critical care are less likely to be comfortable with performing CPR or less familiar with associated guidelines and literature. This may account for some of the discrepancy in compliance among European regions.

The number of board-certified or eligible specialists and residency trainees accounted for ≤4% of respondents from half of the surveyed areas. Interestingly, the 4 regions with the highest proportion of specialists and resident respondents (18–43%) were also the only 4 regions to obtain a total compliance score above 0%. An improvement in RECOVER guideline compliance and awareness has been previously demonstrated when comparing veterinarians with specialist training to those in general practice and our study supports this finding (17, 18, 20). Due to a low response rate, the present study is underpowered to enable subdivision between specialists and general practitioners within each region. Specialty training was statistically significantly associated with increased BLS, ALS, and overall RECOVER compliance, however specialist compliance with CPR guideline recommendations was lower than expected. This may be due to the lack of distinction between type of specialty in this study, but is considered most likely attributed to environmental and individual characteristics, and potentially insufficient guideline distribution or format (21).

While more than 90% of respondents in all surveyed regions offered CPR in their practice, a difference in the perceived importance of good CPR skills existed among regions. The overall median score of importance remained high and the variation noted between countries was likely multifactorial and associated with regional differences in attitudes toward companion animals, and perhaps veterinary training impacting confidence and opinion of individual respondents.

More than half of the survey respondents were familiar with RECOVER guidelines in only four of the eight surveyed regions and perceived adherence to guideline recommendations was regionally different. The discrepancies between perceived and actual compliance with RECOVER guidelines could reflect a lack of understanding surrounding what is truly required to achieve compliance, as well as the concept that awareness of a policy does not necessarily correlate with adequate understanding or knowledge of what is specifically written. The self-reported questionnaire format of data collection also lends itself to obsequiousness bias and social desirability bias, in which responses are provided based on what the respondent believes is expected of them or desired by the investigator (22, 23). It is important to state that self-reported or calculated compliance is not the same as actual CPR performance or knowledge, and may not reflect what occurs in individual clinical scenarios (22).

More concerning was the poor awareness of RECOVER CPR guidelines despite this being the major and most recent resource available for veterinary CPR technique. Dissemination of new information to graduated professionals is a global challenge, particularly to those outside of academic practice. The onus is often on the individual to maintain an awareness of developments in their profession. This is less likely to occur if such information is not readily available in a convenient and readable format. Consensus statements are often considered lengthy and overwhelming and thought should be given to the format of such documents and the provision of a summary of pertinent information (21, 24).

The English language is the commonly accepted language for medical research, however most citizens of the countries in this study do not speak English as a first language, with a moderate proportion of the population not speaking English at all (25). It is feasible to conceive that non-English speaking veterinarians may have difficulty interpreting publications written only in English. This has been demonstrated in several studies evaluating the impact of teaching and testing students in their non-native language (26–28). Psychology students with a predominant language of Dutch, but with 4 years of English teaching, were found to perform at the same level in both languages on a recognition test, but with much lower performance in the English language on a free recall test (26). Similarly, a higher test score was achieved by Arabic students when a scientific comprehension examination was written in a hybrid of English and simplified Arabic, as opposed to English alone (27). Additionally, a group of Chinese students and teachers reported unsatisfactory teaching and reduced class interaction when English was instituted as the medium of instruction (28). Although achieving conceptual equivalence across multiple languages is a challenge, the World Health Organization produces many of their publications in over 70 languages (29). The RECOVER guidelines are now available in English, Spanish, Portuguese, Mandarin and Japanese, however translation into further languages such as German and French may be beneficial. Additionally, our findings suggest that despite these translation efforts, accessibility or dissemination may require further improvement to ensure all veterinarians are aware of and can familiarize themselves with the major resource for veterinary CPR. These may be considerations for implementation as part of future RECOVER guideline updates.

With a reported maximum of 14% respondents in one region, overall compliance with all three aspects of CPR preparedness, BLS, and ALS was poor in all Western European regions. Countries that had the highest overall compliance rate also tended to have the highest awareness of RECOVER guidelines, however Italy had the highest guideline awareness percentage and yet had some of the lowest compliance scores. Respondents from Italy also reported performing CPR more frequently than any other region, with 72% of respondents performing CPR 6 or more times in a year. This may have led to individuals seeking out guidance on CPR technique and therefore an increased awareness of RECOVER guidelines despite this not translating into self-reported compliance.

The poor overall RECOVER guideline compliance demonstrated in some countries (such as Italy, the Netherlands and Spain) despite above average compliance scores in individually evaluated sections was likely influenced by characteristics of the professional, the patient, and the environment resulting in compliance failure when the recommendations are considered as a whole (21).

### Importance of CPR Preparedness, BLS and ALS Compliance

Compliance with RECOVER guideline recommendations in the areas of CPR preparedness, BLS, and ALS were overall low and differed among surveyed regions. Veterinary CPR outcomes vary between institutions but can be improved significantly when teaching and performing CPR in accordance with the RECOVER guidelines (13). A previously proposed chain of survival suggests that in order to see such benefits and successfully discharge as many patients post-CPR as possible, all aspects including early cardiopulmonary arrest recognition, high-quality CPR and adequate post-arrest care, are likely of equal importance (30).

The maximum reported preparedness compliance was found in 35% of GER/AUS respondents in the current survey. In all other regions, preparedness compliance was lower than 30%. The preparedness measures investigated in our survey were those believed to be most important to facilitate prompt recognition of cardiopulmonary arrest, initiation of CPR, and the provision of guideline compliant CPR, all of which have been shown to positively affect CPR patient outcomes (13, 31, 32). The most common factors contributing to preparedness incompliance in our study were not having completed any CPR training in the past 6 months and the lack of a displayed CPR algorithm at respondents' practices. This is similar to factors contributing to preparedness incompliance found in an international survey predominantly answered by North American respondents (17).

While the optimal interval for CPR training in human and veterinary medicine remains undetermined, the RECOVER guidelines currently recommend CPR training every 6 months (1, 7). Several studies in human medicine suggest that training session intervals of 1 to 3 months and the frequent clinical use of CPR skills lead to improved CPR skill retention (33–35). Further information would be needed to fully understand factors precluding bi-yearly CPR training in our surveyed population, but it seems plausible that many respondents did not recently attend CPR training courses because these were not offered in their geographical region. While an effort should be made to increase offerings for veterinary CPR certification courses in Europe, it is important to keep in mind that hands-on CPR training on a smaller scale can and should also be offered to staff by individual hospitals. If such training is performed well and in adherence with RECOVER guideline recommendations, this can significantly increase veterinary CPR patient outcomes (13).

More respondents displayed emergency drug dosing charts than CPR algorithms in their practices even though both cognitive aids are likely valuable to improve adherence to CPR guideline recommendations (1, 7). Even though incompliance rates with display of a CPR algorithm are similar to previous international veterinary CPR surveys, it remains possible that the unavailability of CPR algorithms in the national language deters many respondents from utilizing this tool and efforts to translate and disseminate veterinary CPR cognitive aids should be improved. There is minimal explanation for the lack of a regularly maintained crash box given this can often be created at low cost with minor effort, however lack of awareness for the necessity and benefit of its presence may explain the compliance deficit in these instances. This is corroborated by France, Portugal and Switzerland having the lowest RECOVER awareness among respondents and the highest incompliance surrounding provision of a crash cart.

Both inappropriate positive pressure ventilation rates and chest compression rates for dogs and cats were frequent contributors to BLS incompliance in most surveyed European regions and incorrectly reported chest compression response rates were higher than reported in a previous international survey (17). Hypo- and hyperventilation during CPR should be avoided due to negative effects of hypoxemia and hypercapnia on patient outcomes, and prolonged increases of elevated intrathoracic pressure during hyperventilation decreasing coronary perfusion pressure (1, 8, 36–39). Based on limited experimental evidence in pigs, a ventilation rate of 10 breaths/min is currently recommended during small animal CPR, while rates >20 breaths/min could negatively affect patients' hemodynamic status (1, 8). Due to a lack of evidence on acceptable ventilation rate ranges in dogs and cats, and in accordance with previously published compliance studies by RECOVER guideline authors, a range of 6–15 breaths/min was deemed an acceptable and RECOVER guideline compliant practice in the current survey (1, 17). To optimize cardiac output during CPR, overly low and high chest compression rates should be avoided as they can lead to incomplete chest recoil between compressions, impaired venous return and decreased coronary and cerebral perfusion pressure (40, 41). The most likely factor for inadequately chosen ventilation and chest compression rates in our study is unawareness of most recent veterinary CPR guideline recommendations, which could potentially be improved with wider guideline dissemination as has been shown in North America (20). Once again, those countries with the lowest RECOVER awareness (France, Switzerland, and Portugal) had the highest BLS incompliance.

The majority of respondents in all surveyed regions reported ALS practices incompliant with RECOVER guidelines and the most common factors responsible for ALS incompliance were lack of access to an electrical defibrillator and routine administration of intravenous fluids for volume expansion during CPR, which is similar to incompliance factors reported in an international veterinary CPR survey (17). An electrical defibrillator may be considered specialized equipment and the likely infrequent utilization combined with the moderate expense and training required could reasonably prohibit availability in veterinary hospitals. Donaldson et al. did not use access to a defibrillator as a requirement for ALS compliance and report much higher rates of ALS compliance in North American veterinarians than found in our study (20). Although ALS compliance scores improved in almost all European regions if defibrillator access is excluded from score assignment, guideline compliance remains woefully inadequate. In contrast to people, shockable cardiac arrest rhythms comprise a minority of arrest rhythms in veterinary patients, yet diagnosis of a shockable cardiac arrest rhythm and appropriate defibrillation in dogs has been shown to be associated with a higher likelihood of achieving return of spontaneous circulation (32, 42, 43). Lastly, the routine administration of intravenous fluids during CPR has been reported by previous veterinary CPR surveys (6, 17). Despite uptake of many other RECOVER recommendations by veterinarians in North America, the routine administration of intravenous fluids during CPR is still widely reported. Intravascular volume expansion in euvolemic patients during CPR increases right atrial pressure and decreases myocardial and cerebral perfusion and the knowledge of potentially detrimental effects and importance of selective intravenous fluid administration must be more widely disseminated (44).

### Limitations

Our study has several limitations. Firstly, due to the international nature of the study and numbers of collaborators, electronic distribution of the survey was chosen over postal surveys. Electronic surveys have previously been shown to have lower response rates compared to postal surveys in human medicine and have demonstrated limited response rates in previous veterinary CPR survey projects (6, 17, 18, 45).

Collaborators were identified to each facilitate survey generation and dissemination in one or several Western European countries but survey distribution techniques could not be standardized among countries. Despite our best efforts to disseminate the survey through every countries' respective national veterinary association, not all contacted institutions were responsive to such requests. As a result, our study is likely limited by selection bias. During the translation of questionnaires to the respective countries' national languages, collaborators endeavored to consider cross-cultural differences and adapt and formulate questions that could be understood by veterinarians training and practicing in different professional systems. Despite this effort, ideal cross-cultural adaptation cannot be guaranteed and misinterpretation of questions due to language and cultural differences could have influenced our results (46).

The proportion of residency trained or board-certified specialists responding to the survey differed among surveyed regions. This likely further biased our survey results, as it has previously been shown that veterinarians residency-trained or board-certified in emergency and critical care or anesthesia are more likely to be aware of RECOVER guideline existence and more commonly report adherence to guideline recommendations (17, 18). Due to non-standardized survey distribution and unknown numbers of veterinarians contacted, response rates to our survey could not be reliably determined. Despite the overall satisfactory number of participants, responses remained small for some of the surveyed countries, precluding subgroup analysis such as CPR practices in general practitioners or board-certified specialists.

Survey based research has been used to obtain information for decades, however, more rigorous assessments of this methodology identified the potential to introduce various response biases in all stages from question formatting through to distribution and follow-up (23, 47). Despite best efforts to reduce these, the method of data collection could be considered a limitation of this study.

The timing of survey conduction in the early stages of the COVID-19 pandemic cannot be disregarded. The year 2020 represented a stressful and unprecedented time for small animal medicine with increasing case numbers and limitations in veterinary staffing posed by the pandemic (48, 49). This could not only have influenced response to the survey but also the compliance with RECOVER guideline recommendations as for example attending a veterinary conference and hands-on CPR training was prohibited in many European countries toward the end of the surveyed time period.

Assignment of a compliance score was based on evaluation of factors selected by the authors, and in line with those used in previous CPR guideline compliance surveys (17, 18, 20). A requirement for achieving complete compliance is frequent training and resource availability which are both situational dependent and often outside of the control of the individual respondent. It should be considered that selection of certain factors may bias the compliance score of a country with more limited resource availability. It must also be reiterated that the compliance scores in this study are not directly translatable to quality of clinical practice but simply reflect how closely respondents reportedly adhere to the RECOVER CPR guidance. Nevertheless, extensive evidence was reviewed during the formation of these guidelines and compliance to expert guidelines is known to improve patient outcomes (13–16, 50–54). As such, regardless of circumstance, individual efforts should be made to comply with all areas of the RECOVER guidelines.

Lastly, only a selection of the 2012 RECOVER CPR guidelines were assessed for compliance assessment in this study and by specifically seeking veterinarian responses, only part of the veterinary health care team potentially involved in CPR efforts was surveyed. This approach was chosen to maintain comparability between the current survey and previously conducted veterinary CPR surveys but limits the generalizability of our results with regards to comprehensive CPR knowledge and skills and does not allow us to assess RECOVER guideline awareness more broadly across all veterinary professionals (6, 17, 20).

### Conclusions

In conclusion, awareness of and compliance with RECOVER guideline recommendations varied considerably among Western European regions surveyed. Less than half of the surveyed veterinarians were aware of CPR guideline existence in four of eight regions. Guideline awareness positively influenced self-reported compliance with guideline recommendations but less than one quarter of veterinarians in all surveyed regions were compliant with all aspects of recommended CPR preparedness, BLS and ALS techniques. In a majority of regions, infrequent CPR training and limitations in rescuer environment largely contributed to overall incompliance. When considering the high number of general practitioners that participated in this study and their perceived importance of good CPR skills, efforts to improve RECOVER guideline awareness and compliance should be considered essential. For future updates of the RECOVER clinical CPR guidelines, efforts for guideline translation into multiple languages with wider dissemination may be prudent, and increased CPR training course availability should be considered in Western Europe.

## Data Availability Statement

The original contributions presented in the study are included in the article/Supplementary Materials, further inquiries can be directed to the corresponding author.

## Ethics Statement

The studies involving human participants were reviewed and approved by Vetsuisse Faculty Ethical Review Board, University of Bern, Switzerland. Written informed consent for participation was not required for this study in accordance with the national legislation and the institutional requirements.

## Author Contributions

SNH and AK contributed to conception and design of the study. SNH organized the database and performed the statistical analysis. SPH reviewed the database. SPH and SNH wrote the first draft of the manuscript. All authors assisted with data collection, survey distribution, manuscript revision, read, and approved the submitted version.

## Conflict of Interest

SNH is a member of the RECOVER initiative research committee and served as an evidence evaluator for the RECOVER 2.0 guidelines. RECOVER is a not for profit organization and those services were provided on a volunteer basis. The author will not have any financial gain from increased distribution of CPR guidelines to small animal practitioners. SNH is a certified RECOVER CPR instructor and has led CPR certification workshops in exchange for an honorarium from course organizers such as the American College of Veterinary Emergency and Critical Care and the Veterinary Emergency and Critical Care Society. The remaining authors declare that the research was conducted in the absence of any commercial or financial relationships that could be construed as a potential conflict of interest.

## Publisher's Note

All claims expressed in this article are solely those of the authors and do not necessarily represent those of their affiliated organizations, or those of the publisher, the editors and the reviewers. Any product that may be evaluated in this article, or claim that may be made by its manufacturer, is not guaranteed or endorsed by the publisher.

## Abbreviations

ALS: Advanced life support
BLS: Basic life support
CPR: Cardiopulmonary resuscitation
ECG: Electrocardiogram
ESP: Spain
EtCO_2_: End-tidal carbon dioxide
FRA: France
GER/AUS: Germany and Austria
ITA: Italy
NLD: Netherlands
PRT: Portugal
RECOVER: Reassessment Campaign on Veterinary Resuscitation
SUI: Switzerland
UK/IE: England, Northern Ireland, Scotland, Wales, and Ireland.
