# Supplementary material for: Self-Reported Clinical Practice of Small Animal Cardiopulmonary Resuscitation and Compliance With RECOVER Guidelines Among Veterinarians in Eight Western European Regions
Source: Front Vet Sci. 2022 Jul 14;9:919206. doi: 10.3389/fvets.2022.919206 (PMC9352391; doi:10.3389/fvets.2022.919206)
Supplement: Supplementary file 1 [file Table_1.docx]

**Supplementary Table 1**: List of organizations and social media groups contacted for distribution of electronic survey, dates of initial contact and any subsequent reminders for each participating Western European country.

| ***Country*** | ***Medium of survey distribution*** | ***Initial contact*** | ***Reminder*** |
| --- | --- | --- | --- |
| ***Austria*** | Emails to Austrian Veterinary Medical board, Veterinary Faculty at the national university hospitals and primary email addresses of referral practices identified via the Google^®^ search engine. | December 2019 - January 2020 | None |
|  | Facebook groups of the Veterinary Faculty at the national university hospitals. | December 2019 - January 2020 | None |
| ***France*** | Email to all veterinary surgeons registered with the National Council of the Order of Veterinarians [Conseil national de l'Ordre des vétérinaires]. | December 2019 | None |
| ***Germany*** | Published in electronic newsletter and on homepage of the Society of German Veterinarians (Bundestieraerzteblatt, Grüner Heinrich) | December 2019 - January 2020 | None |
|  | Emails to German Veterinary Medical board, Emergency Departments of the Veterinary Faculties at the national university hospitals, and primary email addresses of referral practices identified via the Google^®^ search engine. | December 2019 - January 2020 | None |
|  | Facebook groups of Veterinary Faculties at the national university hospitals and veterinary Facebook groups. | December 2019 - January 2020 | None |
| ***Ireland*** | Link to survey posted on Veterinary Council of Ireland website homepage | 20^th^ January 2020 | None |
|  | Email to all-staff email address for the national university hospitals. | January 2020 | None |
| ***Italy*** | E-mail to Italian Companion Animal Veterinary Association (Società Culturale Italiana Veterinari per Animali da Compagnia), Italian Veterinary Emergency and Critical Care Society (Società Italiana di Medicina d’Urgenza e Terapia Intensiva Veterinaria), Italian National Veterinary Association (Associazione Nazionale Medici Veterinari Italiani), and Italian Union of Veterinary Societies (Unione Italiana Società Veterinarie) | December 2019 | January 2020 |
|  | Shared on personal Facebook profile of co-author | December 2019 | January 2020 |
|  | E-mail to primary e-mail address of the national university hospitals, and several large veterinary clinics. | December 2019 | January 2020 |
| ***Liechtenstein*** | Email to primary email addresses of referral practices identified via the Google^®^ search engine. | December 2019 - January 2020 | None |
| ***Netherlands*** | Posted in the Facebook group “Het Dierenartsengilde” for the Royal Dutch Society for Veterinary Medicine [Koninkliijke Nederlandse Maatschappij voor Diergeneeskunde]. | Beginning of January 2020 | End of January 2020 |
|  | E-mail to the primary email address of the three referral veterinary practices. | February and March 2020 | None |
| ***Portugal*** | E-mail to listserv for the Portuguese Association of Companion Animals [Associacao Portugesa de Medicos Veterinarios Especialistas em Animais de Companhia] | January 2020 | April 2020 |
|  | Posted in the Facebook group  “Forum veterinario de Portugal” | January 2020 | April 2020 |
| ***Spain*** | Listing in the virtual newsletter and paper magazine distributed by the General Council of Veterinary Colleges of Spain [Consejo General de Colegios Veterinarios de Españna], newsletter of the Spanish Society of Veterinary Anesthesia and Analgesia (Sociedad Española de Anestesia y Analgesia Veterinaria), a regional council of a veterinary colleges, and in Argos Veterinary Medicine Magazine | December 2019 | None |
|  | Shared post on LinkedIn and personal Facebook profile of co-author.  Distributed to Facebook group “Sociedad Española de Anestesia y Analgesia Veterinaria” and “Argos Veterinary Medicine Magazine” | December 2019 | None |
|  | E-mail to the primary email address of the national university hospitals, two referral veterinary practices and fifteen official regional veterinary colleges. |  |  |
| ***Switzerland ^17*^*** | Distribution in electronic news letter of the Society of Swiss Veterinarians (Gesellschaft Schweizer Tieraerzte) and the Swiss Association for Small Animal Medicine (Schweizerische Vereinigung fuer Kleintiermedizin) | July 2019 | August 2019 |
|  | Facebook groups of the Vetsuisse faculty the national university hospitals. | July 2019 | August 2019 |
| ***United Kingdom (including England, Northern Ireland Scotland and Wales)*** | Email to 1056 veterinary practices primary email address (provided by the Royal College of Veterinary Surgeons from their list of registered practices willing to be contacted for research purposes). | January 2020 | None |
|  | Email to national listserv for two national veterinary corporations. | January 2020 | March 2020 |
|  | Email to all-staff email address for the veterinary teaching hospitals associated with universities. | January 2020 | None |
| ***All countries*** | Informal communication and sharing of the survey link to known colleagues/associates in respective countries. | January 2020 | N/a |

* Refer to reference 18 in the main manuscript.
