# Supplementary material for: Self-Reported Clinical Practice of Small Animal Cardiopulmonary Resuscitation and Compliance With RECOVER Guidelines Among Veterinarians in Eight Western European Regions
Source: Front Vet Sci. 2022 Jul 14;9:919206. doi: 10.3389/fvets.2022.919206 (PMC9352391; doi:10.3389/fvets.2022.919206)
Supplement: Supplementary file 2 [file Data_Sheet_1.docx]

**Supplementary data 1** – Full questionnaire uploaded to SurveyMonkey^®^ and link distributed to English-speaking countries. Questions were translated to French, German, Italian, Dutch, Portuguese and Spanish for other participating countries.

1. Do you currently provide any clinical veterinary care to small animals (dogs and/or cats)?
    Yes
    No
2. *Optional (in cases of shared questionnaires):*
   In which country do you currently practice?
3. What is your gender?
    Male
    Female
    Other
    Prefer not to answer
4. What is your age? ________
5. When did you graduate from veterinary school?
   <1 year ago
   1-5 years ago
   6-10 years ago
   11-15yrs ago
   16-20 yrs ago
   >20yrs ago
6. In your veterinary school curriculum, were there lectures or skills laboratories on CPR?
    Yes
    No
7. Which best describes your current professional status?
   General practitioner (including not boarded ER vet)
   FVH
   Board certified specialist
   Student
   Intern
   Resident
   Not currently practicing veterinary medicine
   other
   If “other”, please specify: ___________
8. *Optional (branching algorithm based on question 7 answer):*
   What is the internship in which you are enrolled?
   small animal rotating
   small animal ER
   large animal
   other
9. *Optional (branching algorithm based on question 7 answer):*In which year of your residency are you?
   1^st^
   2^nd^
   3^rd^
   other
10. *Optional (branching algorithm based on question 7 answer):*
    Where are you fulfilling your residency requirements?
    University
    Private practice
11. *Optional (branching algorithm based on question 7 answer):*
    What is the residency in which you are enrolled?
    Anesthesia

Emergency and critical care
Internal medicine
Surgery
Neurology
other

1. *Optional (branching algorithm based on question 7 answer):*
   By which specialty organization are you board certified? ________
2. *Optional (branching algorithm based on question 7 answer):*
   When did you graduate from residency?
   <1 year ago
   1-5 years ago
   6-10 years ago
   11-15yrs ago
   16-20 yrs ago
   >20yrs ago
3. *Optional (branching algorithm based on question 7 answer):*
   Where did you complete your residency?
   University
   Private Practice
4. How many veterinary clinicians work in your current place of employment?
   1
   2
   3
   4
   5
   5-10
   11-15
   16-20
   20-30
   30-40
   40-50
   >50
5. What is the daily average number of dogs and cats that you personally attend to at your clinic?
   None
   1-5
   6-10
   11-15
   16-20
   >20
6. Which of the following best describes the emergency service your clinic provides?
   Emergency service during regular business hours only
   Emergency service during regular business hours and on call after hours
   Emergency service or clinic open after regular business hours only
   Emergency service or clinic open 24h and able to hospitalize patients
   Emergency/Critical Care center with at least one board-certified emergency clinician
   Other ______________
7. Which of the following best describes your workplace?
   Private practice (general or specialty) with no interns or residents
   Private practice with interns
   Private practice with residents
   Private practice with both interns and residents
   University practice
8. Which of the following best describes your clinic’s patient distribution?
   Small animals (including exotics)
   Dogs only
   Cats only
   Mixed with >50% small animals
   Mixed with <50% small animals
   Other__________
9. Approximately what percentage of dogs and cats that you personally see, present as emergencies?
   None
   1-10%
   11-25%
   25-50%
   51-75%
   75-99%
   100%
10. How long has it been since you last participated in veterinary CPR training?
    Never
    >3 years ago
    1-3 years ago
    6mo – 1 year ago
    within the last 6 months
11. Do you offer CPR at your practice?
     Yes
     No
     Only to certain cases
12. If no, what are the reasons?
    I don’t know how to perform CPR
    I don’t have the resources
    My clientele does not wish it/there is no demand for it
    It is too expensive
    Patients are not monitored closely enough
    I don’t ever consider it indicated
    It is pointless/never works
13. If only in specific cases, please specify what cases (e.g. only if arrest occurs under anesthesia) _______
14. Do you routinely ask for a code status when admitting/sedating/anesthetizing patients at your practice?
     Yes
     No
15. How many times per year are you personally involved in performing CPR?
    Never
    1

2-5
6-10
11-20
>20

1. On average, how many people make up the resuscitation team during CPR in your practice?
   1-2
   3
   4
   5
   >5
2. How long do you continue CPR before you consider it futile and pronounce the patient dead?
    No success within 5 minutes
    No success after 5-10 minutes
    No success after 10-15 minutes
    No success after 15-20 minutes
    No success after 20-30 minutes
    No success after >30 minutes
3. Which of the following preparedness measures for CPR are in place in your practice? Check all that apply.
   In house continuing education in veterinary CPR
   Regular training drills for staff likely to be involved in CPR
   Regularly maintained crash cart or crash station
   Emergency drug dosing chart displayed
   CPR algorithm displayed
   Specific CPR record sheet to document the CPR
   None of the above
4. How important do you think the following preparedness measures are?
   In house CE on veterinary CPR:
    1 2 3 4 5 6 7 8 9 10

(not important at all) (absolutely necessary)

Regular training drills for staff likely to be involved in CPR:
 1 2 3 4 5 6 7 8 9 10

(not important at all) (absolutely necessary)

Regularly maintained crash cart or crash station:
 1 2 3 4 5 6 7 8 9 10

(not important at all) (absolutely necessary)

Emergency drug dosing chart displayed:
 1 2 3 4 5 6 7 8 9 10

(not important at all) (absolutely necessary)

CPR algorithm displayed:
 1 2 3 4 5 6 7 8 9 10

(not important at all) (absolutely necessary)

Specific CPR recording sheet to document the efforts:
 1 2 3 4 5 6 7 8 9 10

(not important at all) (absolutely necessary)

1. Which of the following techniques have you employed during CPR?
   Closed chest compressions
   Open chest CPR

Aortic cross clamping
Interposed abdominal compressions
Impedance threshold device (ITD)
None of the above

1. In dogs, with what frequency (compressions per minute) do you perform external chest compressions during CPR?
   <60
   60-80
   80-100
   100-120
   120-150
   150-200
   >200
2. In cats, with what frequency (compressions per minute) do you perform external chest compressions during CPR?
   <60
   60-80
   80-100
   100-120
   120-150
   150-200
   >200
3. By which means have you supported breathing during CPR in dogs or cats?
   Mouth to snout ventilation
   Mask and ambu bag
   Endotracheal intubation
   Emergency tracheostomy
   Supraglottic airway (LMA)

Jen Chung GV 26 acupuncture point stimulation
Other

1. What gas do you routinely use to deliver breaths during CPR?
   Room air

Supplemental oxygen
Exhaled breath

1. In dogs, what ventilation rate (breaths per minute) are you generally targeting during CPR?
   1-5
   6-15
   16-30
   31-45
   45-60
   To match compression rate
   As many as possible
2. In cats, what ventilation rate (breaths per minute) are you generally targeting during CPR?
   1-5
   6-15
   16-30
   31-45
   45-60
   To match compression rate
   As many as possible
3. Which of the following monitoring tools or techniques do you have available at your clinic for use during CPR? Check all that apply.
   ECG
   Capnograph
   Pulse oximeter
   Oscillometric BP measurement
   Doppler BP
   Ultrasound
   Stethoscope
   None of the above
4. Which do you use routinely during CPR?
   ECG
   Capnograph
   Pulse oximeter
   Oscillometric BP
   Doppler BP
   Ultrasound
   Direct pulse palpation
   Palpation of apex beat
   Stethoscope
   MM color and CPR

How important do you think these monitoring techniques are during CPR?

ECG
 1 2 3 4 5 6 7 8 9 10

(not important at all) (absolutely necessary)

Capnograph
 1 2 3 4 5 6 7 8 9 10

(not important at all) (absolutely necessary)

Pulse oximeter
 1 2 3 4 5 6 7 8 9 10

(not important at all) (absolutely necessary)

Oscillometric BP
 1 2 3 4 5 6 7 8 9 10

(not important at all) (absolutely necessary)

Doppler BP
 1 2 3 4 5 6 7 8 9 10

(not important at all) (absolutely necessary)

Ultrasound
 1 2 3 4 5 6 7 8 9 10

(not important at all) (absolutely necessary)

Direct pulse palpation
 1 2 3 4 5 6 7 8 9 10

(not important at all) (absolutely necessary)

Palpation of apex beat
 1 2 3 4 5 6 7 8 9 10

(not important at all) (absolutely necessary)

Stethoscope
 1 2 3 4 5 6 7 8 9 10

(not important at all) (absolutely necessary)

MM color and CRT
 1 2 3 4 5 6 7 8 9 10

(not important at all) (absolutely necessary)

1. Do you have an electrical defibrillator available?
   Yes
   No
2. Which of the following techniques have you employed during CPR?
   External defibrillation
   Internal defibrillation

Precordial thump
None of the above

1. Which of the following drugs do you have readily available at your practice? Check all that apply.
   Atropine
   Epinephrine (Adrenaline)

Vasopressin
Lidocaine
Amiodarone
Sodium bicarbonate
Calcium gluconate
Doxapram
Mannitol
Glucocorticoids
Opioids
Naloxone

Flumazenil
Atipamezole (Antisedan)
Other

1. Which ones have you used during CPR attempts in the last two years?
   Atropine
   Epinephrine (Adrenaline)

Vasopressin
Lidocaine
Amiodarone
Sodium bicarbonate
Calcium gluconate
Doxapram
Mannitol
Glucocorticoids
Opioids
Naloxone

Flumazenil
Atipamezole (Antisedan)
Other

1. Do you routinely use intravascular volume expansion therapy (e.g. crystalloid or colloid bolus) as part of your CPR strategy?
   Yes
   No
2. How frequently have you used the methods listed below for drug administration during CPR?

Intravenous drug administration
 1 2 3 4 5 6 7 8 9 10

(never) (always)

Intraosseous drug administration in a juvenile animal
 1 2 3 4 5 6 7 8 9 10

(never) (always)

Intraosseous drug administration in an adult animal
 1 2 3 4 5 6 7 8 9 10

(never) (always)

Direct (blind) intracardiac injection
 1 2 3 4 5 6 7 8 9 10

(never) (always)

Via endotracheal tube
 1 2 3 4 5 6 7 8 9 10

(never) (always)

1. Conducting effective CPR is an essential skill in clinical small animal veterinary medicine.
    0 ________________________________________________ 100

(Not essential) (absolutely essential)

1. How do you judge your proficiency in performing CPR?
   Poor
   Fair
   Good
   Very good
   Excellent
2. Have you heard of the Reassessment Campaign on Veterinary Resuscitation (RECOVER) CPR guidelines published in 2012?
   Yes
   No
3. Do you practice CPR according to the RECOVER guidelines in your patients?
   Yes
   No
