# Supplementary material for: Self-Reported Clinical Practice of Small Animal Cardiopulmonary Resuscitation and Compliance With RECOVER Guidelines Among Veterinarians in Eight Western European Regions
Source: Front Vet Sci. 2022 Jul 14;9:919206. doi: 10.3389/fvets.2022.919206 (PMC9352391; doi:10.3389/fvets.2022.919206)
Supplement: Supplementary file 3 [file Data_Sheet_2.docx]

**Supplementary data 2** – The questions selected for analysis from the survey distributed to participating countries. Example provided shows question written in English.

1. Do you currently provide any clinical veterinary care to small animals (dogs and/or cats)?
    Yes
    No
2. *Optional (in cases of shared questionnaires):*
   In which country do you currently practice?
3. What is your gender?
    Male
    Female
    Other
    Prefer not to answer
4. What is your age? ________
5. Which best describes your current professional status?
   General practitioner (including not boarded ER vet)
   FVH
   Board certified specialist
   Student
   Intern
   Resident
   Not currently practicing veterinary medicine
   other
   If “other”, please specify: ___________
6. How many veterinary clinicians work in your current place of employment?
   1
   2
   3
   4
   5
   5-10
   11-15
   16-20
   20-30
   30-40
   40-50
   >50
7. What is the daily average number of dogs and cats that you personally attend to at your clinic?
   None
   1-5
   6-10
   11-15
   16-20
   >20
8. Which of the following best describes your clinic’s patient distribution?
   Small animals (including exotics)
   Dogs only
   Cats only
   Mixed with >50% small animals
   Mixed with <50% small animals
   Other__________
9. Approximately what percentage of dogs and cats that you personally see, present as emergencies?
   None
   1-10%
   11-25%
   25-50%
   51-75%
   75-99%
   100%
10. How long has it been since you last participated in veterinary CPR training?
    Never
    >3 years ago
    1-3 years ago
    6mo – 1 year ago
    within the last 6 months
11. Do you offer CPR at your practice?
     Yes
     No
     Only to certain cases
12. How many times per year are you personally involved in performing CPR?
    Never
    1

2-5
6-10
11-20
>20

1. Which of the following preparedness measures for CPR are in place in your practice? Check all that apply.
   In house continuing education in veterinary CPR
   Regular training drills for staff likely to be involved in CPR
   Regularly maintained crash cart or crash station
   Emergency drug dosing chart displayed
   CPR algorithm displayed
   Specific CPR record sheet to document the CPR
   None of the above
2. In dogs, with what frequency (compressions per minute) do you perform external chest compressions during CPR?
   <60
   60-80
   80-100
   100-120
   120-150
   150-200
   >200
3. In cats, with what frequency (compressions per minute) do you perform external chest compressions during CPR?
   <60
   60-80
   80-100
   100-120
   120-150
   150-200
   >200
4. In dogs, what ventilation rate (breaths per minute) are you generally targeting during CPR?
   1-5
   6-15
   16-30
   31-45
   45-60
   To match compression rate
   As many as possible
5. In cats, what ventilation rate (breaths per minute) are you generally targeting during CPR?
   1-5
   6-15
   16-30
   31-45
   45-60
   To match compression rate
   As many as possible
6. Which of the following monitoring tools or techniques do you use routinely during CPR?
   ECG
   Capnograph
   Pulse oximeter
   Oscillometric BP
   Doppler BP
   Ultrasound
   Direct pulse palpation
   Palpation of apex beat
   Stethoscope
   MM color and CPR
7. Do you have an electrical defibrillator available?
   Yes
   No
8. Which of the following drugs do you have readily available at your practice? Check all that apply.
   Atropine
   Epinephrine (Adrenaline)

Vasopressin
Lidocaine
Amiodarone
Sodium bicarbonate
Calcium gluconate
Doxapram
Mannitol
Glucocorticoids
Opioids
Naloxone

Flumazenil
Atipamezole (Antisedan)
Other

1. Do you routinely use intravascular volume expansion therapy (e.g. crystalloid or colloid bolus) as part of your CPR strategy?
   Yes
   No
2. Conducting effective CPR is an essential skill in clinical small animal veterinary medicine.
    0 ________________________________________________ 100

(Not essential) (absolutely essential)

1. Have you heard of the Reassessment Campaign on Veterinary Resuscitation (RECOVER) CPR guidelines published in 2012?
   Yes
   No
2. Do you practice CPR according to the RECOVER guidelines in your patients?
   Yes
   No
